# Real-World Impact of Finerenone on Albuminuria in Patients with Diabetes and CKD

**DOI:** 10.3390/ijms262311584

**Published:** 2025-11-29

**Authors:** Marina López-Martínez, Juan León-Román, Ehimy Suárez, Sara Nuñez-Delgado, María Antonieta Azancot, Jorge Iván Zamora-Carrillo, Marc Patricio-Liébana, Alexander Sánchez Olaya, Irene Agraz, Sheila Bermejo, Laia Sans, Nestor Toapanta, Natalia Ramos, María José Soler

**Affiliations:** 1 Department of Nephrology, Vall d’Hebron University Hospital, Pg. de la Vall d’Hebron, 119, 08035 Barcelona, Spain; juancarlos.leon@vallhebron.cat (J.L.-R.); amy.suarez@vallhebron.cat (E.S.); sara.nunez@vallhebron.cat (S.N.-D.); mariaantonieta.azancot@vallhebron.cat (M.A.A.); jorgeivan.zamora@vallhebron.cat (J.I.Z.-C.); marc.patricio@vallhebron.cat (M.P.-L.); jorgealexander.sanchez@vallhebron.cat (A.S.O.); irene.agraz@vallhebron.cat (I.A.); sheila.bermejo@vallhebron.cat (S.B.); nestor.toapanta@vallhebron.cat (N.T.); natalia.ramos@vallhebron.cat (N.R.); 2 Department of Medicine, Universitat Autònoma de Barcelona, 08193 Barcelona, Spain

**Keywords:** albuminuria, chronic kidney disease, diabetes, finerenone, hyperkalemia

## Abstract

Patients with diabetes and chronic kidney disease (CKD-DM) often have residual albuminuria despite pharmacological treatment. Finerenone targets mineralocorticoid overactivation, but real-world evidence remains limited. This study evaluated the impact of finerenone in a cohort of patients with CKD-DM. This was a real-life study including patients with CKD-DM and an estimated glomerular filtration rate (eGFR) ≥ 20 mL/min/1.73 m^2^, treated with finerenone, aged ≥ 18 years, and followed at the Nephrology Department of Vall d’Hebron Hospital. Clinical and laboratory data were collected at baseline and at 1, 3, and 6 months of treatment. Changes in albuminuria and eGFR were analyzed in patients who completed 6 months of follow-up. A total of 60 patients were included in the analysis; 39 (65%) were male, with a median age of 79 ± 10.12 years. Finerenone was initiated at 10 mg daily in 57 patients (95%), with 38.3% escalating to 20 mg after 1 month. After 6 months, the urinary albumin-to-creatinine ratio (UACR) decreased by 37.1% (*p* = 0.012, n = 34). Patients with an initial eGFR drop > 20% showed a greater UACR decrease of around 43% at 3 (*p* = 0.012) and 6 months (*p* = 0.013). A significant 9.5% decline in eGFR was observed at 1 month, followed by stabilization at 3 and 6 months. Finerenone was discontinued in 10% of the patients due to adverse events. Hyperkalemia occurred in 18.3% of the patients, but no hospitalizations for adverse events or heart failure were reported. In summary, finerenone induced a significant 37.1% reduction in albuminuria after 6 months of treatment. This reduction was more pronounced in patients who experienced an initial eGFR dip ≥ 20%. Finerenone was generally well tolerated and appears to be a promising therapeutic strategy for reducing albuminuria in this population.

## 1. Introduction

Chronic kidney disease (CKD) and diabetes are two closely related diseases. Around 40% of patients with diabetes develop CKD. In addition, patients with CKD are also at risk of developing diabetes and cardiovascular events. According to the World Health Organization, 332 million people were living with diabetes and CKD in 2022 [1]. Both diseases increase mortality rates, currently causing over 2 million deaths per year [1].

The pathophysiology of CKD in persons with diabetes mellitus (CKD-DM) involves metabolic factors such as hypertension, altered tubuloglomerular feedback, and mineralocorticoid (MR) overactivation [2]. Together, these events cause inflammation and oxidative stress, hyperfiltration, and, finally, CKD [3]. 

As glomerular filtration rate (GFR) is typically estimated in clinical practice, hyperfiltration goes unnoticed [4,5]. Albuminuria is the usual biomarker used to diagnose kidney damage secondary to diabetes. Indeed, albuminuria is one of the most critical determinants of CKD progression. Moreover, it has recently been recognized as one of the five cardiovascular risk factors [6]. Even when GFR remains above 90 mL/min/1.73 m^2^, the presence of albuminuria increases the risk of mortality [7]. Remarkably, the increase in mortality begins at 10 mg/g of albuminuria and escalates to nearly four times that of a healthy individual at 1 g/g of albuminuria [7]. Therefore, albuminuria is one of the main kidney outcomes in clinical trials, representing one of the most significant therapeutic targets in CKD-DM.

Notably, some new drugs have demonstrated an ability to slow CKD progression over the last decade. Adding sodium-glucose transporter inhibitors (SGLT2is) and/or glucagon-like peptide-1 receptor agonists (GLP-1RAs) to the traditional renin–angiotensin system inhibitors (RASis) has been shown to reduce albuminuria [8]. These antiproteinuric drugs have significantly delayed the progression of CKD and improved survival prognoses [9,10,11]. Despite these novel treatments, most patients with CKD-DM still have residual albuminuria, perpetuating cardiovascular risk and CKD [2].

Interestingly, special attention should be paid to finerenone, the most recently approved drug for CKD-DM treatment. This molecule fills the gap in the treatment of MR overactivation. Its high selectivity for MR makes finerenone much more effective at reducing albuminuria than classical MR antagonists such as eplerenone and spironolactone [12]. The FIDELITY study, which combined the results of the FIGARO and FIDELIO trials, demonstrated a significant reduction in the composite kidney outcome in patients treated with finerenone. Moreover, finerenone achieved a significant reduction of 30–40% in albuminuria after 3–4 months of treatment [13,14,15,16,17].

Finerenone was approved and commercialized in Spain in May 2024. Although clinical trials and studies have predicted a promising and synergistic effect of finerenone [18,19], few results from real-world clinical practice have been published. In our opinion, such an analysis is particularly important in the local context as it bridges the gap between clinical trials and daily clinical practice. The current study aims to evaluate the tolerability of finerenone after its initiation in a real-world cohort of patients with CKD-DM, as well as its short-term effects on renal function and albuminuria over a 6-month follow-up period.

## 2. Results

### 2.1. Baseline Characteristics of the Studied Population

A total of 60 patients who initiated finerenone treatment and met all the inclusion criteria were included in this study. None of them met the exclusion criteria. Follow-up data were available for all patients at 1 month, for 41 (68.3%) at 3 months, and for 34 (56.7%) at 6 months. The progressive reduction in the number of patients across follow-up visits was attributable to the timing of finerenone initiation relative to the study closure, as some patients had not completed the 3- or 6-month follow-up by the end of the data collection. No patients were lost to follow-up or excluded after baseline. Baseline patient characteristics are shown in Table 1. All patients had diabetes, with a median HbA1c of 7.2% [6.4–7.6]; 39 (65%) were male, and the mean age was 79 ± 10.12 years. The median eGFR was 49.49 [33.5–60.5] mL/min/1.73 m^2^, mean potassium 4.54 ± 0.49 mmol/L, and median albuminuria 479 [189.5–1199.5] mg/g (Table 1). The main comorbidities were hypertension (98.3%), dyslipidemia (90%), and obesity (38.8%), with 96.7% of the patients meeting the criteria for metabolic syndrome (Table 1). Of these patients, 34 fulfilled the criterion of 6 months of follow-up. The mean age of this subgroup was 69.15 ± 8.16; 67.6% were male, and the median eGFR was 42 [27.75–58.25] mL/min/1.73 m^2^, with an albuminuria value of 485 [231.75–1118.50] mg/g (Table 1).

### 2.2. Renoprotective Treatment During the Study

**Figure 1:**
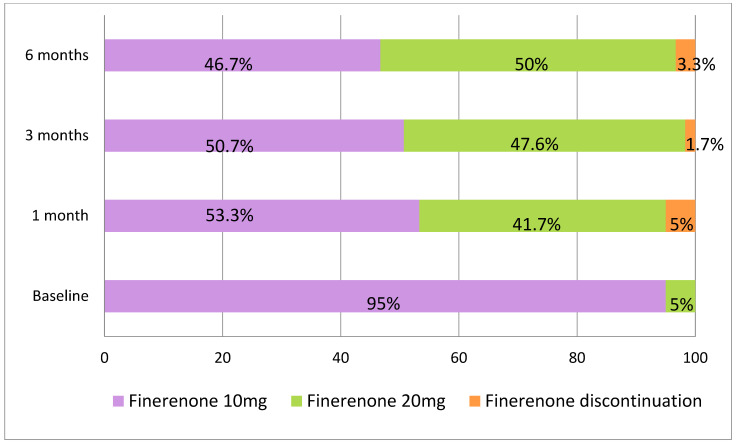
Finerenone dosing at baseline, 1 month, 3 months, and 6 months and discontinuation rates across patients.

At baseline, 55 patients (91.7%) were receiving RASi treatment, 51 (85%) patients were on SGLT2is, and 26 (43.3%) patients were receiving GLP-1RAs (Table 1). Finerenone was initiated at a dose of 10 mg once daily in 57 patients (95%) (Figure 1). The dose was uptitrated to 20 mg in 23 patients (38.3%) at the 1-month follow-up visit; thus, a total of 41.7% of the patients were treated with 20 mg of finerenone (Figure 1). At the 3-month follow-up, 42 patients were assessed. In two additional cases, the finerenone dose was increased to 20 mg, resulting in 47.6% of the patients receiving the 20 mg dose at that time (Figure 1). Among the 34 patients with a 6-month follow-up, the dose was escalated in 3 more patients, bringing the total to 50% receiving the 20 mg dose by the end of the follow-up period (Figure 1). 

Finerenone was discontinued in three patients (5%) during the first month as a result of acute kidney injury; in one of these cases, it was also associated with hyperkalemia. It was discontinued in one patient (1.7%) at 3 months due to palliative status and in two patients (3.3%) at 6 months due to hyperkalemia (Figure 1). The drug was reintroduced after three months at a dose of 10 mg in three patients in whom finerenone had been discontinued. At the time of reintroduction, the mean serum creatinine was 2.08 ± 0.34 mg/dL and the mean serum potassium was 4.41 ± 0.42 mmol/L. All three patients were followed for an additional three months, and renal function remained stable (mean creatinine 2.00 ± 0.53 mg/dL) without hyperkalemia (mean potassium 4.91 ± 0.26 mmol/L).

### 2.3. eGFR and Albuminuria Evolution During Follow-Up

**Figure 2:**
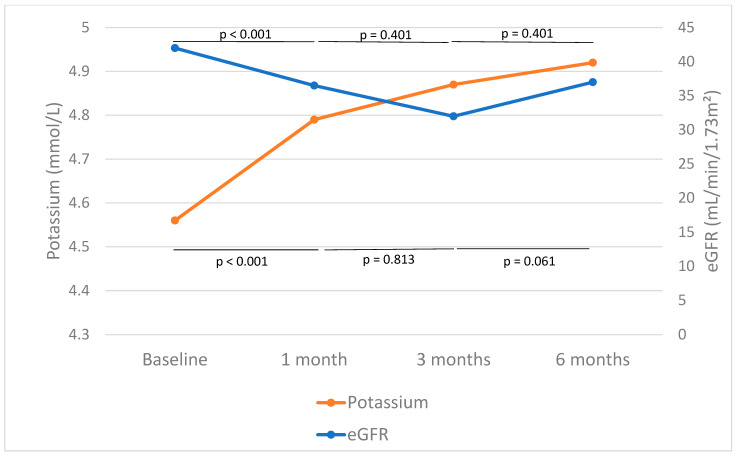
Effect of finerenone on estimated glomerular filtration rate (eGFR) and potassium. The median and mean values of eGFR and potassium before and after 1, 3, and 6 months of finerenone initiation are shown in blue and orange, respectively. The horizontal lines in the graph represent the *p*-values for the intervals of changes in eGFR. The lines at the bottom of the figure show *p*-values related to changes in serum potassium across the same intervals.

The first analysis included the whole group with 1 month of follow-up, during which time eGFR dropped from 49.49 [33.50–60.50] to 44.80 [28.25–58.00] mL/min/1.73 m^2^ (*p* < 0.001). The subgroup analysis with 6 months of follow-up revealed an initial eGFR drop at 1 month, from 42 [27.75–58.25] to 36.50 [23.75–53.00] mL/min/1.73 m^2^ (*p* < 0.001) (Figure 2, Table 2). No significant differences were observed when compared to the values at 3 months (*p* = 0.401) or 6 months (*p* = 0.371), suggesting stabilization of renal function following the initial eGFR drop (Figure 2).

**Figure 3:**
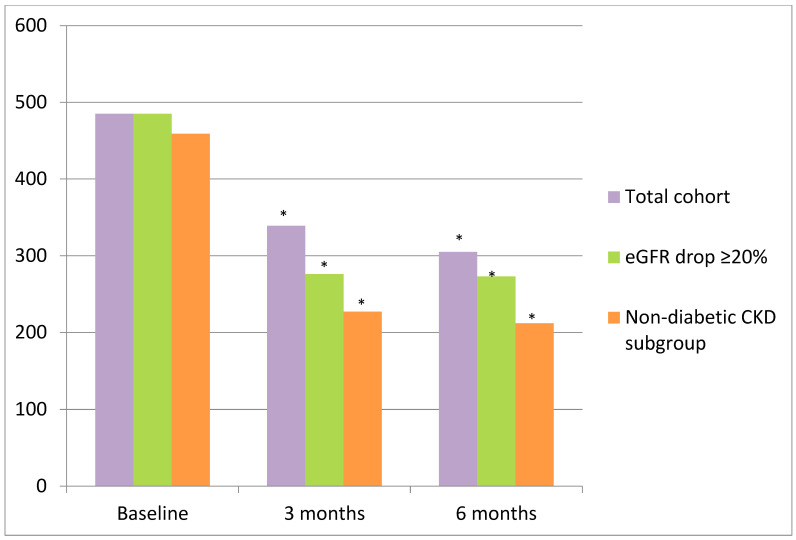
Evolution of albuminuria after finerenone initiation in the total cohort, subgroup with eGFR drop > 20%, and non-diabetic CKD subgroup. Percentage in red represents the change in UACR from baseline. (*) *p* < 0.05.

The relative decrease in UACR was of 30.1% (*p* = 0.012) and 37.1% (*p* = 0.012) after 3 and 6 months of finerenone treatment, respectively (Figure 3, Table 2). A reduction in albuminuria of more than 30% was observed in 70.57% of individuals after 6 months of follow-up. The modification of UACR during the first month was not assessed due to the limited number of patients with available data. In the subgroup of patients with an eGFR drop of >20% (n = 10) in the first month, there was a reduction in albuminuria to 43.09 (*p* = 0.012) and 43.71% (*p* = 0.013) after 3 and 6 months of treatment, respectively (Table 3, Figure 3). In the subgroup of patients with a BMI ≥ 30 Kg/m^2^ (n = 16) at baseline who were followed-up for 6 months, albuminuria decreased from 467.50 (203.75–940.25) mg/g to 276.00 (54.00–1606.00) mg/g after 3 months (*p* = 0.953) and to 186 (67–1843) mg/g after 6 months (*p* = 0.918) of treatment.

Of these patients, 15 (35.71%) had CKD that was not only attributed to DM and also met the condition of 6 months of follow-up (Table 4). In this group of patients, albuminuria also decreased from 459 [201–722] mg/g to 227 [55–2167] mg/g at 3 months (*p* = 0.037) and to 212 [67–1900] at 6 months (*p* = 0.041) after finerenone initiation (Figure 3).

### 2.4. Other Clinical Parameters

In the entire cohort with 1 month of follow-up, potassium levels increased from 4.55 ± 0.48 to 4.78 ± 0.49 mmol/L (*p* < 0.001). When the subgroup with 6 months of follow-up was evaluated, potassium levels increased significantly after 1 (*p* = 0.003), 3 (*p* = 0.003), and 6 months (*p* < 0.001) of treatment (Figure 2, Table 2). It is noteworthy that, when comparing potassium levels after 1 month of finerenone treatment with subsequent measurements, no further significant increases were observed. Levels rose from 4.79 ± 0.52 to 4.87 ± 0.45 mmol/L between months 1 and 3 (*p* = 0.813) and from 4.87 ± 0.45 to 4.92 ± 0.42 mmol/L between months 3 and 6 (*p* = 0.061), suggesting stabilization of potassium after the early increase (Figure 2).

The analysis of all clinical parameters in patients with 6 months of follow-up is depicted in Table 2. There was a significant improvement in HbA1c levels from 7.40% to 7.00% (*p* = 0.019) and to 7.05% (*p* = 0.049) at 3 and 6 months of follow-up, respectively. 

### 2.5. Adverse Events

Adverse events are shown in Table 5. Hyperkalemia occurred in 13 patients, all of whom were classified as having mild hyperkalemia (<6 mmol/L), and only 3 cases resulted in finerenone being withdrawn, while the rest were controlled through dietary adjustments, the use of potassium chelators, and the maintenance of finerenone at 10 mg. There were no cases of gynecomastia. There were no hospital admissions due to adverse effects of finerenone or heart failure, and no deaths were reported during the study follow-up.

## 3. Discussion

Our study confirmed the positive effect of finerenone on albuminuria in real-life patients with diabetes and CKD. Renal function remained stable after the initial eGFR dip throughout the follow-up period, and finerenone was generally well tolerated, with only three cases of drug withdrawal due to hyperkalemia. 

Finerenone was initially prescribed at a daily dose of 10 mg for 95% of patients, with 38.3% increasing to 20 mg after 1 month (Figure 1). By 3 months, 47.6% of patients were taking 20 mg daily, and by 6 months, 50% had reached this dose (Figure 1). This gradual dose escalation reflects good patient tolerance and the ability to escalate the dose in a controlled manner highlights the flexibility of the treatment regimen. Unfortunately, there is no data on finerenone dose escalation in the FIDELIO and FIGARO trials [14,17,20]. In real-world practice, the goal is to continue with finerenone and increase the dose where possible, rather than discontinuing treatment, while taking measures to prevent hyperkalemia [21].

As expected, UACR was significantly reduced by 37.4% (Figure 2). These results resemble the albuminuria reduction seen in already published in clinical trials on finerenone [13,16,17], which translates into a better cardiovascular prognosis and survival after 6 months of finerenone. In the FIDELIO-DKD trial [16], which enrolled over 5700 patients with type 2 diabetes and CKD, finerenone achieved a 31% of reduction in UACR as compared with placebo after 4 months, and this reduction was maintained for 36 months. Importantly, finerenone significantly reduced the composite kidney outcome (kidney failure, ≥40% eGFR decline, or renal death) by 18% (HR 0.82; 95% CI 0.73–0.93) and cardiovascular events by 14% (HR 0.86; 95% CI 0.75–0.99) [16,17]. It has also been reported that an early decrease in UACR of ≥30% from baseline serves as a surrogate marker for long-term renal and cardiovascular health [16]. Therefore, since 70% of the patients included in the 6-month follow-up achieved the aforementioned UACR reduction of ≥30%, the improvement in CKD prognosis and cardiovascular risk was significantly enhanced. The FIDELITY analysis [22], a prespecified pooled analysis of FIDELIO-DKD and FIGARO-DKD including more than 13,000 patients, confirmed a significant reduction in both cardiovascular and renal outcomes. There was a 23% lower risk of kidney failure or a sustained ≥57% eGFR decline and a 14% lower risk of major cardiovascular events. Moreover, reports of synergistic and additive effects of finerenone with SGLT2is [19] and GLP1As have also been published [18].

MR overactivation plays a crucial role in the development and progression of all causes of CKD. Secondary hyperaldosteronism promotes hypertension and oxidative stress and reduces Klotho protein production. All of these events lead to inflammatory and fibrotic processes in the kidneys [2,23]. The prompt reduction in albuminuria observed after 3–4 months of finerenone treatment [16,17] cannot be solely attributed to the natriuretic effects of MR antagonists. In fact, the beneficial effects of finerenone on the kidneys have been partially attributed to the amelioration of mitochondrial dysfunction [24] and endothelial damage [25,26], resulting in an anti-inflammatory effect. In cases in which CKD was not only attributed to DM, the reduction in albuminuria was of 53.81% after 6 months of finerenone initiation (Figure 3). This observation supports the hypothesis that finerenone exerts antifibrotic and anti-inflammatory actions beyond CKD-DM. In this regard, the ongoing FIND-CKD trial (NCT05047263)—the first phase 3 study on finerenone in patients with non-diabetic CKD—has recently completed recruitment. Once published, its results are expected to confirm whether the renal benefits observed in CKD-DM patients extend to individuals with CKD without DM. This would potentially expand the clinical indications of finerenone.

Changes in eGFR were also evaluated in this study. A significant 13.3% reduction in eGFR was observed after one month of treatment. However, this was an expected and transient phenomenon. Subsequently, eGFR remained stable between months 1 and 3 and showed a tendency towards recovery between months 3 and 6 (Figure 2), which is consistent with findings reported in the literature. This eGFR dip has also previously been observed after the initiation of RAS inhibitors [27,28,29] and SGLT2is [30,31], reflecting the improvement in excessive intraglomerular pressure. Acute eGFR decline after finerenone initiation is an effect that has already been described and is associated with a reduction in cardiovascular and CKD progression in long-term follow-up [20,22]. Data from trials show that this decline is reversible upon treatment discontinuation and may actually be beneficial for long-term kidney health by slowing disease progression [17,20]. Recent results from the FINEARTS-HF trial suggest that an initial decline in eGFR with treatment initiation does not negate the long-term cardiovascular and renal benefits and may offer protective effects [32]. Indeed, when the eGFR dip was of >20% in the first month, albuminuria decreased by 43.09% and 43.71% after 3 and 6 months of treatment, respectively (Figure 2, Table 3). Therefore, this initial eGFR drop should be recognized once again as a protective effect and a clinical benefit.

In terms of adverse events, finerenone was generally well tolerated. Although potassium levels increased significantly, only 13 of the initial 60 patients (21.67%) experienced mild hyperkalemia (Table 5), and finerenone had to be withdrawn in only 6 patients (10%) (Figure 1). There were no hospital admissions or reported deaths either (Table 5). The incidence of adverse events during treatment was similar in the finerenone and placebo groups in published trials [14,17]. Similarly, hyperkalemia occurred in 18% of patients in the finerenone group in the FIDELIO trial, but no fatal cases were reported [17].

The limitations of our study include its retrospective design, the small number of patients included, and the absence of a control group for comparison. These limitations restrict the generalizability of the conclusions, so the results should be interpreted with caution. However, the fact that we followed patients up for 6 months and that this is one of the first pieces of real-life evidence on finerenone means that we can obtain useful information for daily clinical practice.

The synergistic effect of new antiproteinuric drugs has revolutionized the paradigm of CKD treatment. Our results support the conclusion that finerenone reduces albuminuria by almost 40% after 6 months of treatment, initially causing a drop in eGFR, which subsequently stabilizes in patients with CKD-DM. Moreover, the decrease in UACR after finerenone treatment appears to be more pronounced in patients with an initial eGFR dip of ≥20% compared to their baseline. Finerenone was generally well tolerated, with few and minor adverse events. The observed albuminuria reduction and tolerability align with evidence from controlled trials, reinforcing finerenone’s role as a cornerstone in the multifactorial management of CKD associated with diabetes. Targeting one of the main pathways involved in CKD pathophysiology, such as mineralocorticoid overactivation, with finerenone has proven to be an effective therapeutic strategy. Minimizing albuminuria, which in turn reduces cardiovascular and CKD progression, has become reality in the new era of CKD-DM therapies. However, given the early decline in eGFR observed, finerenone should be used with caution, particularly in patients with advanced CKD. Therefore, finerenone is not recommended for use in patients with an eGFR <25 mL/min/1.73 m^2^. For patients with an eGFR between 25 and 59 mL/min/1.73 m^2^, treatment should be started at a lower dose (10 mg), with close monitoring of serum potassium and eGFR one month afterwards. 

## 4. Materials and Methods

### 4.1. Study Design and Patients

This is a retrospective, observational, longitudinal cohort study conducted in a real-world clinical setting to evaluate the tolerability and short-term effects of finerenone on renal function and albuminuria in patients with CKD and type 2 diabetes. All patients were followed up at the Nephrology Department of Vall d’Hebron Hospital. They were aged at least 18 years and had an albuminuria value of at least 30 mg/g, eGFR by Chronic Kidney Disease Epidemiology Collaboration (CKD-EPI) of >20 mL/min/1.73 m^2^, and a potassium level of <5.5 mmoL/L. An additional inclusion criterion was adequate blood pressure control at baseline, defined as a systolic blood pressure between 110 and 130 mmHg and a diastolic blood pressure between 60 and 80 mmHg. Exclusion criteria included patients younger than 18 years old, those with albuminuria of less than 30 mg/g, eGFR by Chronic Kidney Disease Epidemiology Collaboration (CKD-EPI) ≤ 20 mL/min/1.73 m^2^, or a serum potassium level of ≥5.5 mmol/L. Additionally, patients with inadequate blood pressure control at baseline, defined as a systolic blood pressure below 110 mmHg and/or above 130 mmHg, or a diastolic blood pressure below 60 mmHg and/or above 80 mmHg, were excluded. Patients with clinical conditions contraindicating finerenone use, such as a recent episode of acute kidney injury, severe comorbidities that were incompatible with study participation, incomplete follow-up, or insufficient medical record data were also excluded. The Ethical Committee of Vall d’Hebron University Hospital approved the study protocol EOM(AG)033/2021(5838). Given the retrospective and observational nature of this study, informed consent was not required from the participants. All data were collected from existing medical records, and patient confidentiality was preserved in accordance with institutional and national ethical guidelines.

### 4.2. Study Follow-Up and Data Collection

The study follow-up period started when finerenone was initiated and continued for up to 6 months. At baseline, the following data were collected: age, gender, height, weight, body mass index (BMI), smoking habits, hypertension, diabetes mellitus, chronic obstructive pulmonary disease (COPD), heart failure, hemoglobin, ferritin, hemoglobin A1c (HbA1c), glucose, renal function as assessed by serum creatinine and eGFR (calculated using CKD-EPI equation), serum albumin, sodium, potassium, cholesterol, triglycerides, UACR, treatment with ACEis or ARB, treatment with SGLT2is or GLP1 analogs, and dose of finerenone during the follow-up. Similar variables were collected at follow-up, at 3 months and 6 months. Additionally, adverse events related to finerenone were also assessed. The starting dose of finerenone was determined based on the patient’s eGFR. All patients with CKD-EPI < 60 mL/min/1.73 m^2^ initiated treatment with finerenone at a dose of 10 mg daily. For patients with CKD-EPI > 60 mL/min/1.73 m^2^, the starting dose was determined at the discretion of the treating physician, based on individual patient considerations. We conducted an analysis of the effect of finerenone on drug tolerability, eGFR, and serum potassium one month after its initiation. This initial analysis included all patients from our center who met the inclusion criteria. Subsequently, we performed a separate analysis focusing on the effect of finerenone on laboratory parameters in a subgroup of patients with data available for six months (n = 34). Acute kidney injury was defined as an increase in creatinine of ≥0.3 mg/dL or by ≥1.5 times from baseline. Hyperkalemia was defined as a serum potassium level of ≥5.5 mmol/L. It was categorized according to severity as mild (5.5 to 5.9 mmol/L), moderate (6.0 to 6.4 mmol/L), or severe (≥6.5 mmol/L).

### 4.3. Statistical Analysis

The data were first tested for normal distribution using the Kolmogorov–Smirnov test. Variables exhibiting normal distribution were expressed as means ± standard deviations (SDs). Non-parametric variables such as albuminuria were expressed as medians [interquartile ranges (IQRs): 25th–75th percentiles] and categorical variables were expressed as percentages. To assess how the variables changed over time, we compared each follow-up measurement to the baseline (time 0) within the same group. For non-normally distributed variables, the Wilcoxon signed-rank test for paired samples was used. For variables with a normal distribution, the paired t-test was applied. A *p* < 0.05 was considered statistically significant. Statistical analyses were performed using the SPSS program (version 20, SPSS, Chicago, IL, USA). 

## Figures and Tables

**Table 1:** Clinical characteristics of the patients included in this study.

Clinical Characteristics	Group with 1 Month of Follow-Up (n = 60)	Group with 6 Months of Follow-Up (n = 34)
Age, mean (years)	79 ± 10.12	69.15 ± 8.16
Sex, n (men,%)	39 (65)	23 (67.6)
Smoker (%)	15 (25)	10 (29.4)
Obesity, n (%)	23 (38.3)	16 (47.1)
BMI, median (Kg/m^2^)	29.32 ± 5.67	30.23 ± 5.33
Diabetes mellitus, n (%)	60 (100)	34 (100)
HbA1c, median (%)	7.20 [6.40–7.60]	7.40 [6.60–7.70]
Hypertension, n (%)	59 (98.3)	34 (100)
Dyslipidemia, n (%)	54 (90)	30 (88.2)
Hypertriglyceridemia, n (%)	30 (50)	18 (52.9)
Metabolic syndrome, n (%)	58 (96.7)	33 (97.1)
COPD, n (%)	9 (15)	7 (20.6)
Heart failure, n (%)	11 (18.3)	7 (20.6)
Creatinine, mean (mg/dL)	1.50 ± 0.52	1.63 ± 0.55
eGFR, median (mL/min/1.73 m^2^)	49.49 [33.50–60.50]	42 [27.75–58.25]
Potassium (mmol/L)	4.55 ± 0.48	4.56 ± 0.47
Albuminuria, median (mg/g)	479.50 [189.50–1199.50]	485 [231.75–1118.50]
RAS inhibitors, n (%)	55 (91.7)	31 (91.20)
ARNi, n (%)	5 (8.3)	4 (11.80)
SGLT2i, n (%)	51 (85)	32 (94.10)
GLP1a, n (%)	26 (43.3)	17 (50)

**Table 2:** Changes in clinical parameters with finerenone.

	Baseline	3 Months	*P*	6 Months	*p*
**Hb (g/dL)**	13.36 ± 1.69	13.05 ± 1.78	0.846	13.43 ± 1.63	0.693
**Ferritin (ng/mL)**	115.67 ± 97.93	120.15 ± 101.62	0.193	166.28 ± 215.29	0.149
**HbA1c (%)**	7.40 [6.60–7.70]	7.00 [6.50–7.50]	**0.019**	7.05 [6.30–7.60]	**0.049**
**Glucose (mg/dL)**	133 ± 32.45	125.54 ± 33.34	0.434	133 ± 36.12	0.938
**Cholesterol (mg/dL)**	204.71 ± 301.69	148.15 ± 35.26	0.278	147.47 ± 36.20	0.274
**Triglycerides**	201.44 ± 178.98	206.81 ± 161.87	0.729	187.100.79	0.596
**Creatinine (mg/dL)**	1.63 ± 0.55	1.90 ± 0.53	**0.003**	1.76 ± 0.68	**0.007**
**eGFR** **(mL/min/1.73 m^2^)**	42 [27.75–58.25]	32 [25.25–41.75]	**0.003**	37 [25.00–55.50]	**0.020**
**Sodium (mmol/L)**	140.91 ± 3.01	140.50 ± 4.01	0.170	140.79 ± 2.84	0.802
**Potassium (mmol/L)**	4.56 ± 0.47	4.86 ± 0.45	**0.003**	4.88 ± 0.43	**<0.001**
**UACR (mg/g)**	485 [231.75–1118.50]	339 [124–1338]	**0.012**	305.50 [99.5–1510]	**0.012**

Patients with 6-month follow-up.

**Table 3:** Effect of finerenone on albuminuria (mg/g) according to initial drop in eGFR of ≥ 20% or <20%. Red arrows: percentage in albuminuria decrease compared to baseline (Month 0).

	Albuminuria at Month 0	Albuminuria at 3 Months	*p*	Albuminuria at 6 Months	*p*
**Drop in eGFR ≥20%**	n = 10 485 [380–2479.50]	n = 9 276 [73–1572] ↓ 43.09%	**0.012**	n = 10 273 [134–1066] ↓ 43.71%	**0.013**
**Drop in eGFR <20%**	n = 24 514 [193.50–1044.75]	n = 16 437.50 [156.50–1402.50] ↓ 14.88%	0.301	n = 24 341.50 [75.25–1770.00] ↓ 33.56%	0.179

Patients with 6-month follow-up. *p* < 0.05 was considered statistically significant.

**Table 4:** Etiologies of chronic kidney disease.

CKD-DM (%)	27 (64.29)
Other concomitant etiologies:	15 (35.71)
Reduced nephron mass (%)	2 (13.4)
Nephroangiosclerosis (%)	8 (53.3)
Chronic interstitial nephritis (%)	3 (20)
MCD (%)	1 (6.7)
FGN (%)	1 (6.7)

Patients with 6-month follow-up. MCD: minimal change disease; FGN: fibrillar glomerulonephritis.

**Table 5:** Adverse events during finerenone treatment.

Adverse Events	1 Month n = 60	3 Months n = 41	6 Months n = 34
Gynecomastia (%)	0 (0)	0 (0)	0 (0)
Hypotension (%)	1 (1.7)	0 (0)	0 (0)
Hyperkalemia (%)	6 (10)	3 (10.34)	4 (11.76)
AKI stage 1 (%)	13 (21.67)	8 (27.59)	6 (17.65)
Hospital admissions due to finerenone complications (%)	0 (0)	0 (0)	0 (0)
Hospital admissions for heart failure (%)	0 (0)	0 (0)	0 (0)
Deaths	0 (0)	0 (0)	0 (0)

Patients with 1, 3, and 6 months of follow-up. AKI: acute kidney injury.

## Data Availability

The original contributions presented in this study are included in the article. Further inquiries can be directed to the corresponding authors.
